# Accessory and cavitated uterine masses: a case series and review of the literature

**DOI:** 10.3389/frph.2023.1197931

**Published:** 2023-08-17

**Authors:** S. Dekkiche, E. Dubruc, M. Kanbar, A. Feki, M. Mueller, J-Y. Meuwly, P. Mathevet

**Affiliations:** ^1^ Gynaecology Department, Department Women-Mother-Child, Lausanne University Hospital (CHUV), Lausanne, Switzerland; ^2^ Institute of Pathology, Lausanne University Hospital (CHUV), Lausanne, Switzerland; ^3^ Institut de Recherche Expérimentale et Clinique (IREC), Université Catholique de Louvain, Brussels, Belgium; ^4^ Department of Obstetrics and Gynaecology, HFR Fribourg Hôpital Cantonal, Fribourg, Switzerland; ^5^ Department of Obstetrics and Gynaecology, University Hospital of Berne and University of Berne, Berne, Switzerland; ^6^ Department of Diagnostic and Interventional Radiology, Lausanne University Hospital (CHUV), Lausanne, Switzerland; ^7^ Faculty of Biology and Medicine (FBM), University of Lausanne, Lausanne, Switzerland

**Keywords:** ACUM, Müllerian anomalies, uterine malformations, dysmenorrhea, chronic pelvic pain

## Abstract

**Objectives:**

The purpose of this study is to report nine patients of young women who underwent a surgical treatment of an accessory and cavitated uterine mass (ACUM) in our hospital between 2014 and 2022 and review all cases described in the literature.

**Material and methods:**

The principal outcomes measured are the imaging techniques used to determine the diagnosis, the type of surgery used and the post-operative evolution of symptoms. We also report and analyse the 79 patients found in the literature since 1996 in addition to our 9 patients.

**Results:**

Surgical excision is the only long-lasting treatment. Small invasive surgery with laparoscopic access is the gold standard and most widely used (83.0%). Some new therapeutic procedures have been recently described of which ethanol sclerotherapy seems very promising. Post-operatively, 54.5% of patients have a complete relief of symptoms. MRI is the best imaging technique to identify ACUM. Finally, we refine the description of this pathology and give a more precise definition of it.

**Conclusion:**

Through our literature review and the analysis of our cases, we want to underline an important diagnostic criterion of this pathology: the fallopian tube on the homolateral side of the ACUM never communicates with the latter. It is a capital element for differential diagnosis.

## 1. Introduction

Accessory and cavitated uterine mass (ACUM) is a rare Müllerian duct anomaly of unknown incidence, which affects young women. Since its first description by Cullen in 1908 (1), different terminologies have been used to describe the same entity: juvenile or isolated cystic adenomyoma (2), uterus-like mass or accessory uterine cavity (3) and adenomyotic cyst or cystic adenomyosis (4). In 2010 Acién et al. (5) suggested the term accessory and cavitated uterine mass as a new terminology and defined it by the presence of a non-communicating accessory uterine mass located in the myometrium or within the broad ligament, close to the round ligament insertion, with an otherwise normal genital and urinary tract (3, 5). A list of the diagnostic criteria for ACUM as suggested by Acién et al. is presented in Table 1.

**Table 1:** Diagnostic criteria for accessory and cavitated uterine mass.

Diagnostic criteria for ACUM [as suggested by Acién et al. (5)]
(1) An isolated accessory cavitated mass
(2) Normal uterus (endometrial cavity), tubes and ovaries
(3) Surgical excised mass with pathological examination
(4) Accessory cavity lined by the endometrial epithelium with glands and stroma
(5) Chocolate-brown-coloured fluid content
(6) No adenomyosis (if the uterus removed), but there could be small foci of adenomyosis in the myometrium adjacent to the accessory cavity

While most clinical manifestations for ACUM are non-specific, dysmenorrhea, which ranges from mild to severe, is reported as being the most common symptom. It typically starts soon after menarche and rapidly increases in severity thereafter. Chronic pelvic pain (CPP) and dysfunctional uterine bleeding are also frequent.

ACUM symptoms, such as dysmenorrhea and CPP, are often primarily or secondarily resistant to common analgesics and to classical hormonal treatment as progestogen-only pill (POP), combined oral contraceptive pill (COC) or gonadotropin-releasing hormone agonist (GnRH agonist), as it is the case with endometriosis.

According to Acién and his group, this anomaly required a separate classification and definition from the ESHRE 2013 consensus on congenital malformations of the female genital tract (6) as it does not include this anomaly. At the time of writing, it is considered as part of the unclassified uterine malformations (U6 class).

In their opinion, the origin of this uterine anomaly could be caused by a gubernaculum dysfunction during the embryogenesis expressed through a duplication and persistence of the ductal Müllerian tissue at the attachment level of the round ligament (7).

Our study objectives are (i) to describe nine new patients that we operated, (ii) to do a literature review starting from 1996 and (iii) to analyse and describe this rare pathology as precisely as possible in order to help with the differential diagnosis.

## 2. Materials and methods

We report on nine patients with ACUM treated in Lausanne in Switzerland. All of the patients gave their written consent for the care provided. The study was retrospective, based on medical file analysis, and the standard treatment for this pathology was performed. The written informed consent was obtained from the individuals’ and minors’ legal guardian for the publication of any potentially identifiable images or data included in this article.

For histological analysis, specimens were fixed in 10% neutral-buffered formalin (6–72 h). Formalin-fixed paraffin-embedded samples from specimens were stained with haematoxylin and eosin (HE) (Ventana HE 600 system). Immunohistochemistry (IHC) was performed with an anti-CD10 (56C6, mouse monoclonal, Ventana) antibody using the Ventana BenchMark automated stainer and revealed by the ultraView DAB detection kit (ref. 760-500).

Our literature review aimed to identify all reported cases of this pathology. The following terms were used to search the Medline database using PubMed: juvenile cystic adenomyoma (JCA), uterus-like mass, accessory uterine cavity, adenomyotic cyst, cystic adenomyosis and ACUM. Only the cases corresponding to Acién et al.'s diagnostic criteria of ACUM (5) were included. We found a total of 79 patients between 1996 and April 2020 to which we add our 9 patients. All authors declare no conflict of interest.

## 3. Results

### 3.1. Nine case descriptions

Nine patients who presented with ACUM were operated in our clinic between 2014 and 2022. Their characteristics are described in Table 2. The average age at the time of surgery was 22 years (range 17–35 years).

**Table 2:** Characteristics of nine Swiss patients.

	Age	Age at menarche	Gestity (G)/parity (P)	Year of the operation	Medical/surgical background	Ethnicity
Patient 1	23	13	G1P0	2021	Medical abortion	Caucasian
Patient 2	19	13	G0P0	2022	—	Caucasian
Patient 3	18	12	G0P0	2014	Hypermenorrhoea	Caucasian
Patient 4	22	13	G1P0	2016	Personality disorder, anxiety addiction to cannabis	Caucasian
Patient 5	35	14	G0P0	2018	—	Caucasian
Patient 6	18	12	G0P0	2020	—	Caucasian
Patient 7	30	N/A	G0P0	2021	—	Caucasian
Patient 8	17	12	G0P0	2017	Raynaud syndrome	Caucasian
Patient 9	18	12	G0P0	2017	—	Caucasian

**Figure 1:**
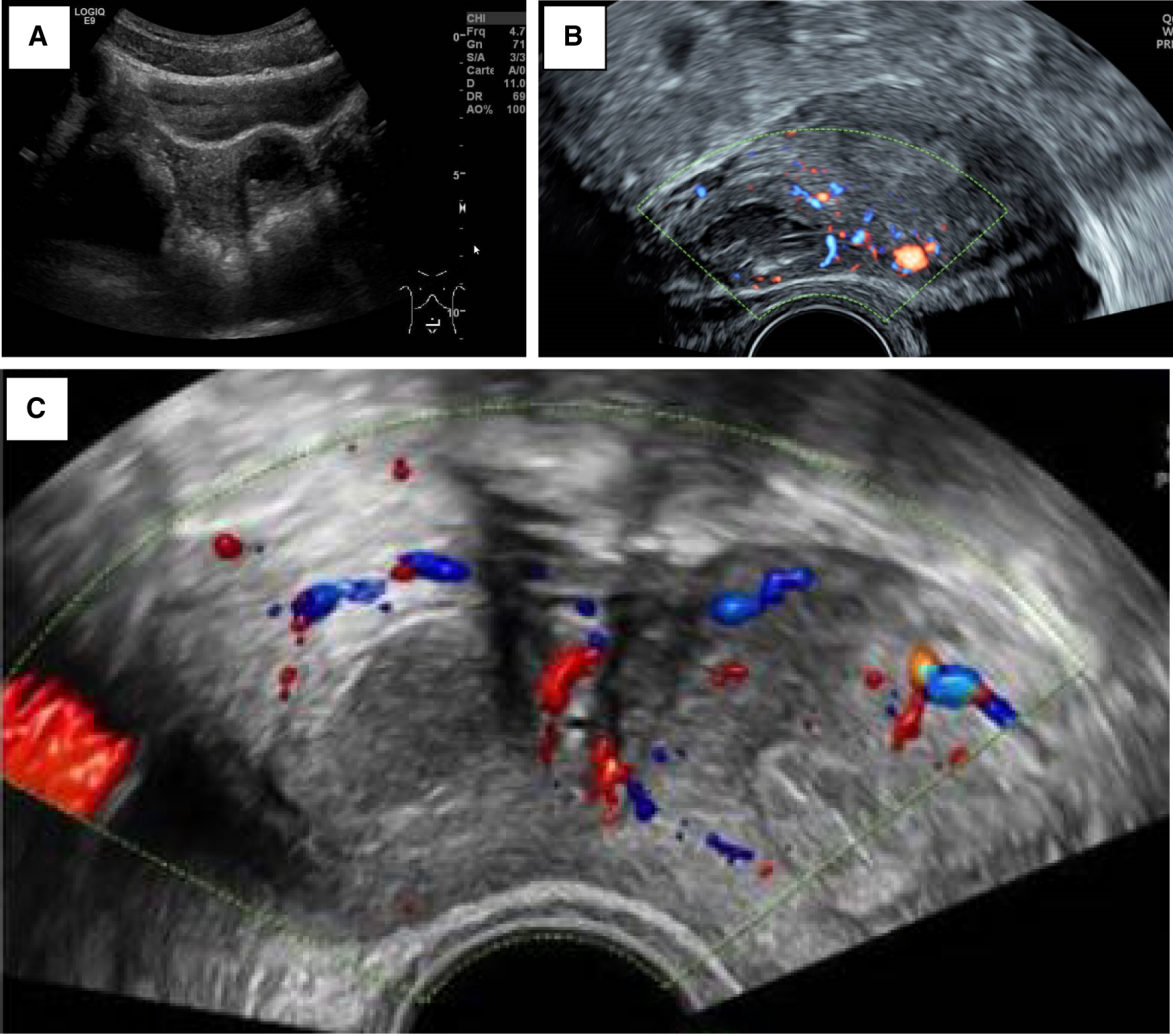
(**A**) Patient 3_TAUS shows a left antero-fundic sub-serous mass of 3.8 cm × 3.6 cm. (**B**) Patient 4_axial plan of TVUS showing the right-lateralized mass separated from the normal uterine cavity by a thick myometrial wall. (**C**) Patient 5_axial echography showing a round right-lateralized mass with hypoechogenic content surrounded by a ring-shaped vascularized capsule.

Severe dysmenorrhea (*n* = 5) and CPP (*n* = 4) were the most common presenting symptoms. As part of the clinical workup, the patients first underwent a pelvic ultrasound (Figure 1). A single lateralized intra-myometrial accessory cavity located under the insertion of the round ligament was found in all patients. The capsule of the lesion had the same echogenicity as the normal myometrium, and the content appeared as hypoechogenic.

**Figure 2:**
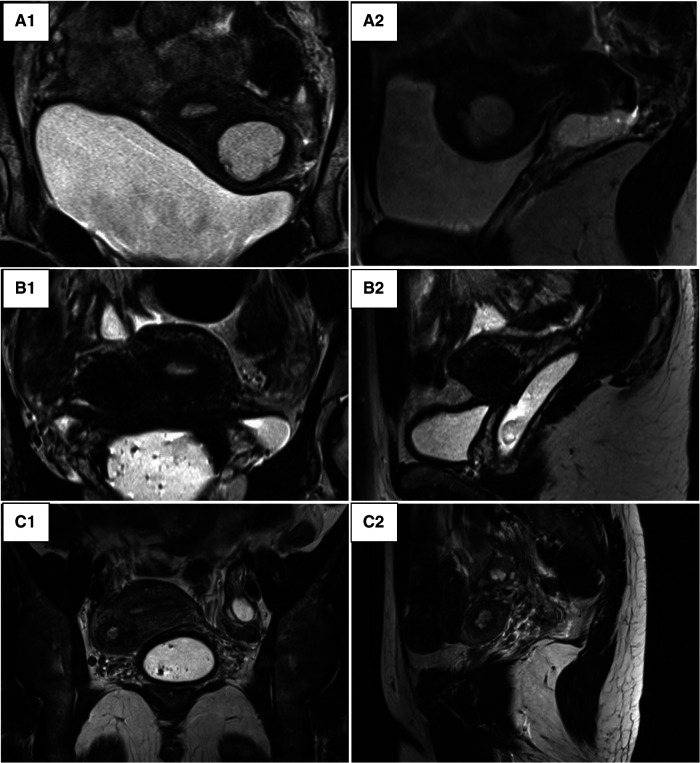
Pelvic MRI. (**A**) Patient 3_round mass in the left anterior myometrial wall suggestive of an accessory endometrial cavity within. (**A1**) T2-weighted coronal image. (**A2**) T2-weighted left lateral sagittal cut. (**B**) Patient 4_a round mass in the right anterior myometrial wall. (**B1**) T2-weighted coronal image. (**B2**) T2-weighted right lateral sagittal cut. (**C**) Patient 5_round mass in the right anterior myometrial wall. (**C1**) T2-weighted coronal image. (**C2**) T2-weighted right lateral sagittal cut.

In addition to an ultrasound, all patients in our series underwent an MRI in order to have a precise description of the lesion (Figure 2). The lesion always had the same characteristics: the mass was isolated and composed of an external thick ring which had the same signal intensity as the junctional zone and regular boundaries. Its contents had a spontaneously hyper-intense signal on T1, T1 fat sat and T2-weighted images speaking for a haemorrhagic material. The rest of the genital and urinary tract was normal across all nine patients.

**Figure 3:**
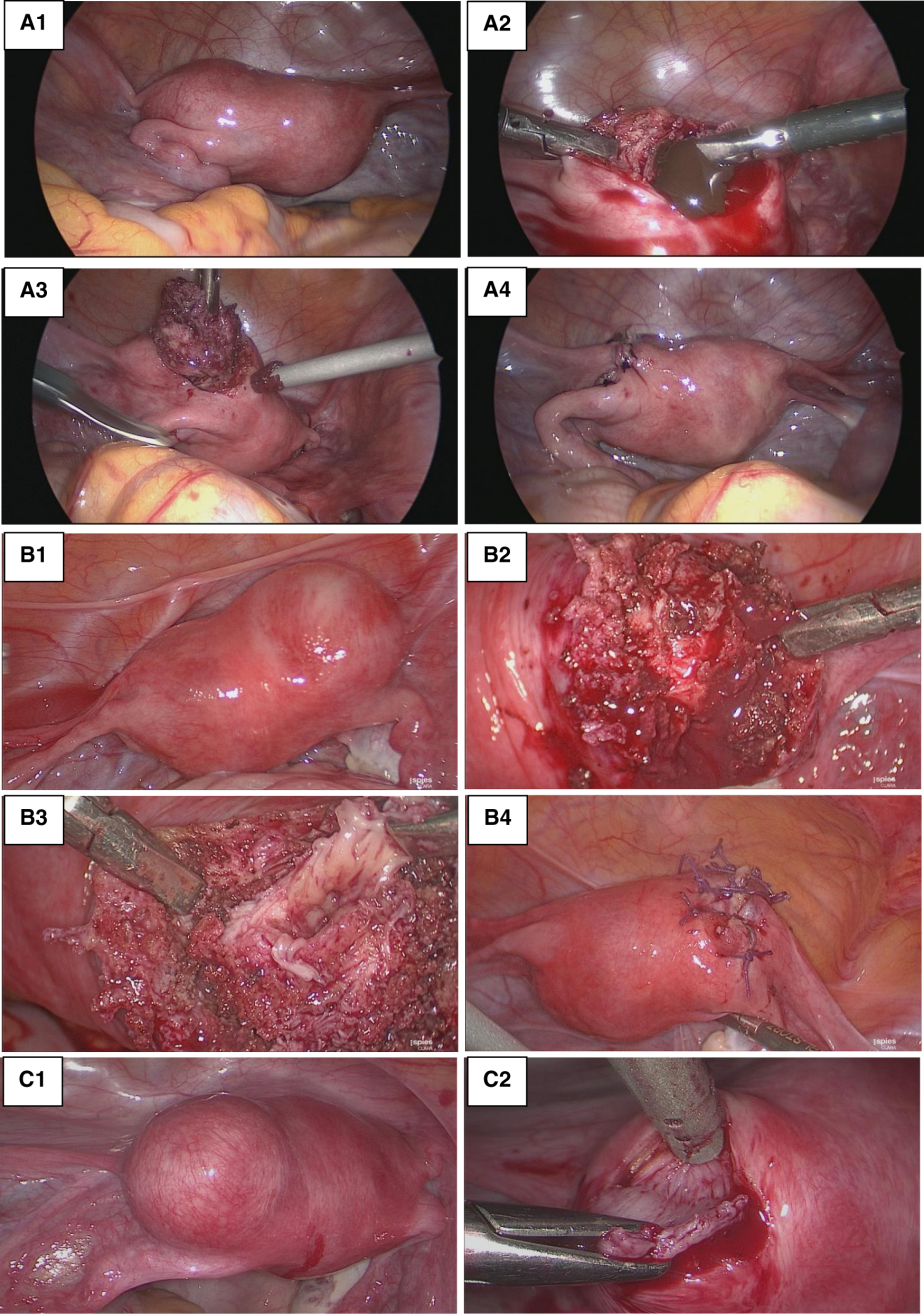
Laparoscopic resection. (**A**) Patient 3_(**A1**) Uterine left-sided mass bulging into the anterior part of the broad ligament under the insertion of the round ligament. (**A2**) Incision of the mass draining chocolate-brown fluid. (**A3**) Excision of the lesion wall. (**A4**) Myometrial defect sutured. (**B**) Patient 5: (**B1**) Right ACUM. (**B2**) Incision of the mass. (**B3**) Excision of the cyst wall with a view of the cystic cavity. (**B4**) Myometrial defect sutured. (**C**) Patient 6: (**C1**) Left ACUM. (**C2**) Excision of the lesions wall.

**Figure 4:**
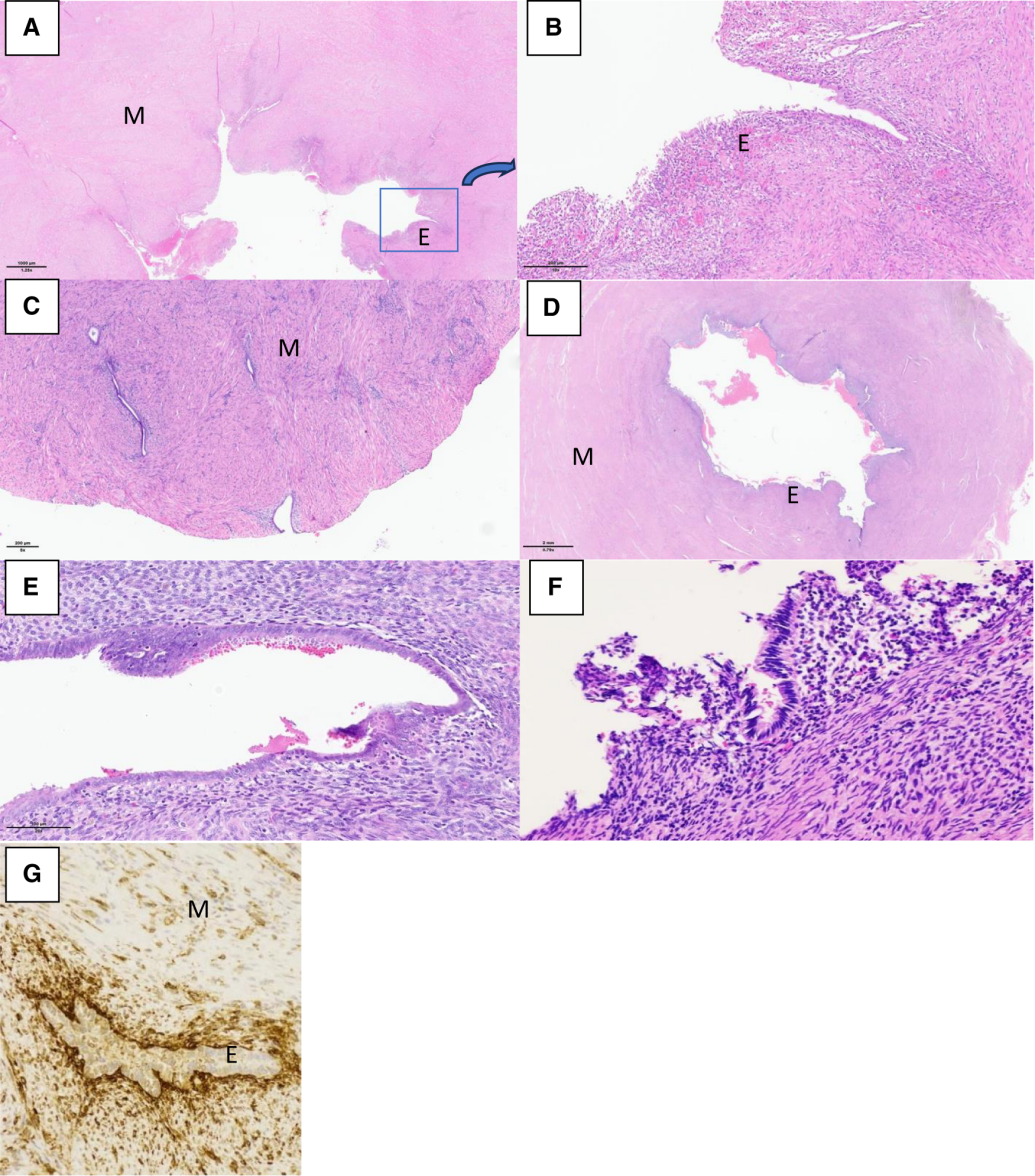
Histological sections with haematoxylin and eosin (HE) stain and immunohistological section showing the ACUM with endometrial epithelium “E”, surrounded by myometrium “M”. (**A**) Patient 1_HE × 1.25. (**B**) Patient 1_HE × 10, zooming in on the highlighted as found in exhibit A. (**C**) Patient 2_HE × 5. (**D**) Patient 3_HE × 0.79. (**E**) Patient 3_HE × 20. (**F**) Patient 5_HE × 5. (**G**) Patient 5_CD10 × 200 immunohistochemistry positivity confirming the presence of endometrial stroma.

The same laparoscopic resection technique was used by four surgeons on all patients (Figure 3). Eight were performed by standard laparoscopy, whereas one of them was performed by a robotic-assisted approach. For the standard laparoscopies, we did a four-trocar approach. The upper abdomen, ovaries and fallopian tubes were macroscopically unremarkable in every patient. The uteruses were deformed by a mass bulging into their anterior part under the insertion of the round ligament. An incision was performed over the swelling zone on the uterus in order to remove the lesion. The progressive dissection around the mass was difficult due to the absence of a correct dissection plan. The average operative time was 128 min (range 80–240 min). No uterine cavity was opened during the procedures. No intraoperative or post-operative complication occurred except for one patient where a fundal uterine perforation by the manipulator occurred. After surgery, the patients were discharged between day 1 and day 3. In all patients, microscopic examination showed a cystic cavity lined by thin endometrium lining and stroma (Figure 4). The myometrial capsule contained small adenomyotic foci. Complementary IHC analysis was performed in patient number 5 to help for diagnosis. Anatomopathology confirmed the initial diagnoses of ACUM in all nine patients.

**Figure 5:**
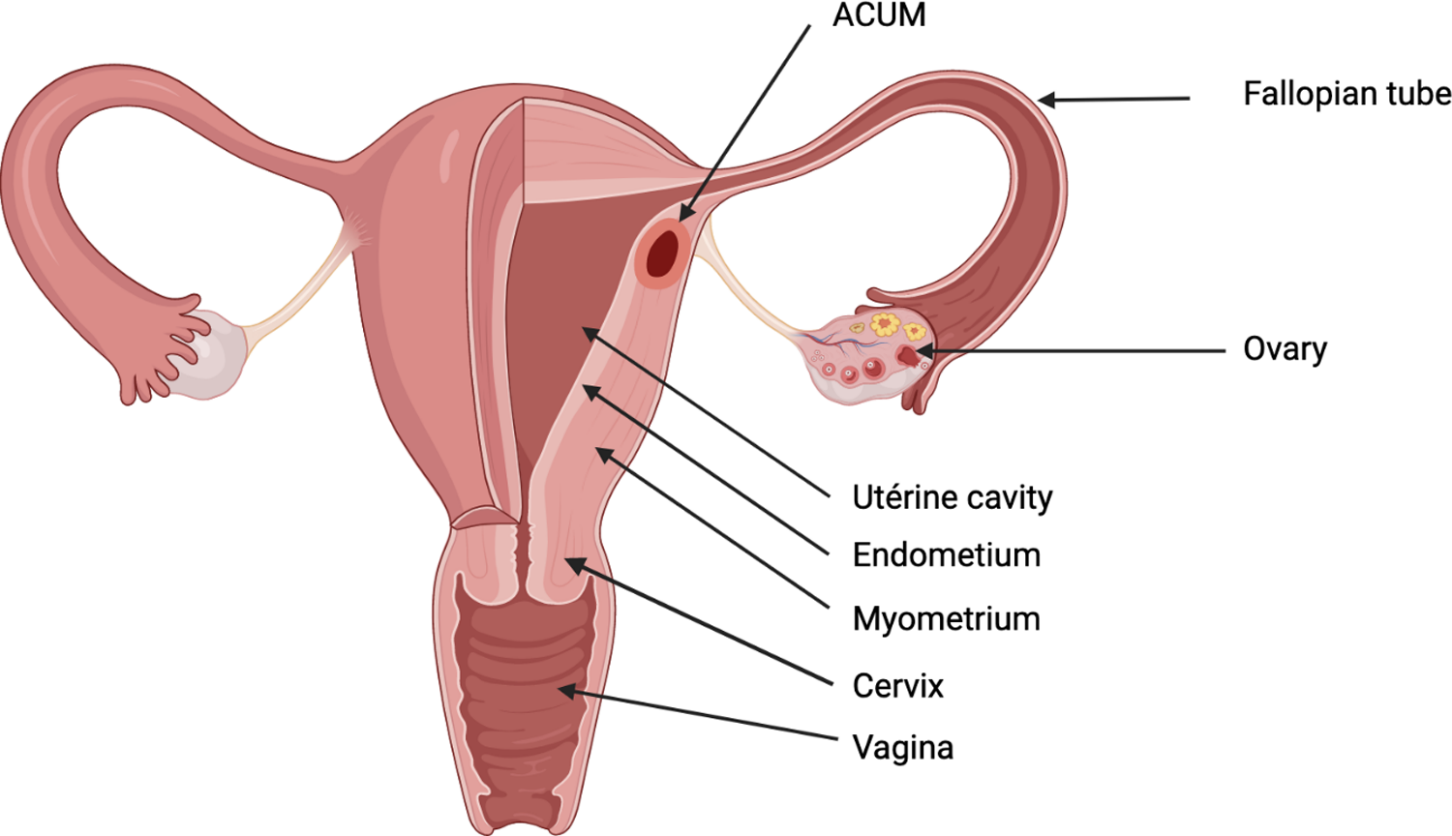
Schematic representation of the location of the left ACUM.

The schematic representation of the location of an ACUM in the reproductive tract is shown in Figure 5 (created with BioRender.com).

### 3.2. Literature review

The characteristics of the 79 patients retrieved from the literature and our nine patients are presented in Table 3. The mean age at diagnosis is 21.9 years (range 14–39 years).

**Table 3:** Review of the 79 published patients of ACUM in the literature and our 9 patients.

References	Number of cases	Age (year)	Major symptom	Investigations	Lesion location	Lesion size (cm)	Type of surgery	Diagnosis	Follow-up (month)
Tamura et al. (8)	1	16	Severe dysmenorrhea	TAUS	Left side of the uterus	3	Laparotomy	Adenomyotic cyst	Diminishing symptoms
				MRI					
				Pyelography					
Potter et al. (9)	1	15	Dysmenorrhea	TVUS	Left side of the uterus anterior to the round ligament	4	Laparotomy	Non-communicating accessory uterine cavity	Symptoms disappeared (12 m)
				Pyelography					
				HSG: normal					
Nabeshima et al. (10)	1	19	Severe dysmenorrhea	TVUS	Right side of the uterus	3	Laparoscopy	Cystic adenomyoma	Symptoms disappeared
				MRI					
				HSG: normal					
Kamio et al. (11)	1	23	Severe dysmenorrhea	TVUS	Left side of the uterus anterior to the round ligament	3	Laparotomy	Adenomyotic cyst	Symptoms disappeared
				MRI					
				HSG: normal					
Takeda et al. (2)	2	20	Severe dysmenorrhea	TVUS	Right side of the uterus anterior to the round ligament	3	Laparoscopy	JCA	Symptoms disappeared
				MRI					
				HSG: normal					
				Pyelography: normal					
		20	Severe dysmenorrhea	TVUS	Left anterior uterine corpus caudal to the round ligament	2.6	Laparoscopy	JCA	Symptoms disappeared
				MRI					
				Pyelography: normal					
Wang et al. (12)	1	26	Severe dysmenorrhea	TVUS	Right anterior uterine horn	3.2	Laparotomy	Cystic adenomyoma	Symptoms disappeared (10 m)
Nabeshima et al. (13)	1	27	Severe dysmenorrhea	TVUS	Right anterior uterine corpus	2	Laparoscopy	Cystic adenomyoma	Symptoms disappeared (12 m)
				MRI					
Ho et al. (14)	1	16	Chronic pelvic pain	TAUS	Right anterior uterine corpus	/	Laparoscopy	Cystic adenomyoma	N/A
				MRI					
Ball et al. (4)	1	19	Dysmenorrhea	TVUS	Left uterine fundus, caudally to the round ligament	2	Laparoscopy	Cystic adenomyosis	Symptoms disappeared (18 m)
				Hysteroscopy: normal					
Acien et al. (5)	4	15	Severe chronic pelvic pain	TVUS	Right anterior uterine wall at the level of the round ligament insertion	3.5	Laparotomy	ACUM	Symptoms disappeared
				HSG: normal					
				Pyelography: normal					
		21	Severe dysmenorrhea	TVUS	Left anterior uterine wall at the level of the round ligament insertion	3	Laparotomy	ACUM	Symptoms disappeared (18 m)
				HSG: normal					
		33	Chronic pelvic pain	TVUS	Right anterior uterine wall at the level of the round ligament insertion	3	Laparoscopy	ACUM	Symptoms disappeared
				HSG: normal					
		32	Severe dysmenorrhea	TVUS	Right anterior uterine wall at the level of the round ligament insertion	5	Total hysterectomy (procedure?)	ACUM	Symptoms disappeared (12 m)
Tijani et al. (15)	1	35	Chronic pelvic pain	TAUS	Left posterior fundus	2.1	Laparotomy	Uterus-like mass	N/A
Liang et al. (16)	1	17	Severe dysmenorrhea	TAUS	Left broad ligament	4.3	Laparotomy	Uterus-like mass	Symptoms disappeared (18 m)
				CT					
Takeuchi et al. (17)	9	25.2	9/9 Dysmenorrhea	9/9 TVUS	Lateral wall near the uterine round ligament attachment site. Right 6/9. Left 3/9	3.2	Laparoscopy	JCA	Diminishing symptoms (35.9 m ± 21.4 m)
				9/9 MRI					
				9/9 Pyelography: normal					
				4/9 HSG: normal					
Akar et al. (18)	1	15	Severe dysmenorrhea	CT	Right lateral wall of the uterus	4.76	Robot-assisted laparoscopy	JCA	N/A
				TVUS					
Chun et al. (19)	1	19	Severe dysmenorrhea	MRI	Left fundus	3	Laparoscopy	JCA	Diminishing symptoms (12 m)
				Pyelography: normal					
Kriplani et al. (20)	4	16	Severe dysmenorrhea	MRI	Right uterine wall near fundus	3.8	Laparoscopy	Cystic adenomyosis	Symptoms disappeared (24 m)
		18	Severe dysmenorrhea	MRI	Right uterine wall	4.2	Laparoscopy	Cystic adenomyosis	Diminishing symptoms (20 m)
		16	Severe dysmenorrhea	MRI	Anterior myometrium	3.1	Laparoscopy	Cystic adenomyosis	Symptoms disappeared (14 m)
		24	Severe dysmenorrhea	MRI	Right uterine wall	3	Laparoscopy	Cystic adenomyosis	Symptoms disappeared (12 m)
Acien et al. (21)	4	36	Chronic pelvic pain	TVUS	Left anterior uterine horn	5	Laparotomy (hysterectomy)	ACUM	Symptoms disappeared
		20	Chronic pelvic pain	TVUS	Left anterior uterine wall below the insertion of the round ligament	4	Laparotomy	ACUM	Symptoms disappeared
				MRI					
				HSG: normal					
		18	Chronic pelvic pain	TRUS	Left anterior horn below the insertion of the round ligament	2.6	Laparotomy	ACUM	Symptoms disappeared (2 m)
				MRI					
		19	Chronic pelvic pain	TVUS	Left anterior horn below the insertion of the round ligament	2	Laparotomy	2×ACUM	Symptoms disappeared
				HSG: normal					
Kumakiri et al. (22)	1	20	Severe dysmenorrhea	TVUS	Anterior side of the uterine body	3	Laparoscopy	JCA	Diminishing symptoms (3 m)
				MRI					
Bedaiwy et al. (23)	1	16	Severe dysmenorrhea	TAUS	Left uterine wall	3	Laparoscopy	ACUM	Symptoms disappeared (9 m)
				MRI					
Jain et al. (24)	1	24	Severe dysmenorrhea	TAUS	Right anterior uterine wall, below the insertion of round ligament	4	Laparoscopy	ACUM	Diminishing symptoms
				MRI					
Paul et al. (25)	3	19	Chronic pelvic pain	TAUS	Right anterior uterine wall, below the insertion of round ligament	2	Laparoscopy	ACUM	Symptoms disappeared (1 m)
				MRI					
		17	Dysmenorrhea	TVUS	Posterior wall of the uterus	4	Laparoscopy	ACUM	Diminishing symptoms (36 m)
		25	Dysmenorrhea	TAUS	Right uterine cornua	3.1	Laparoscopy	ACUM	Diminishing symptoms (84 m)
Pontrelli et al. (26)	1	27	Severe dysmenorrhea	TVUS	Posterior uterine wall	7.5	Hysteroscopy	Giant cystic adenomyoma	Symptoms disappeared (12 m)
				MRI					
Garofalo et al. (27)	1	17	Severe pelvic pain	TVUS	Right anterior uterine wall at the level of the round ligament insertion	1.7	Laparoscopy	ACUM	Symptoms disappeared
				TRUS					
				MRI					
Shen et al. (28)	1	37	Severe dysmenorrhea	MRI	Left uterine wall near the cornua	10	Laparoscopy	Cystic adenomyoma	Symptoms disappeared (6 m)
Dadhwal et al. (29)	1	23	Severe dysmenorrhea	TAUS	Right anterior uterine wall near the cornua	3.9	Laparoscopy	JCA	Symptoms disappeared (12 m)
	1	16	Severe dysmenorrhea	TAUS	Left uterine wall near	4	Laparoscopy	JCA	Symptoms disappeared
				MRI	below the insertion of the round ligament				
Peters et al. (30)	2	19	Dysmenorrhea	TVUS	Left uterine wall	3	Laparoscopy	ACUM	N/A
				MRI					
		39	Pelvic pain	TVUS	Left uterine wall	2.3	Laparoscopy	ACUM	N/A
				MRI					
Strelec et al. (31)	1	14	Severe dysmenorrhea	TAUS	Right uterine wall	4	Laparoscopy	JCA	N/A
Peyron et al. (3)	11	21	Severe dysmenorrhea	MRI (11)	7/11 Left	2.8	Laparoscopy	ACUM	Symptoms disappeared (23 m (range 6–27 m)
			Chronic pelvic pain		4/11 Right				
Park et al. (32)	2	14	Severe dysmenorrhea	TRUS	Right horn of the uterus	3	Laparoscopy	ACUM	Symptoms disappeared (24 m)
				MRI					
		25	Severe dysmenorrhea	TVUS	Left uterine wall	3	Laparoscopy	ACUM	Symptoms disappeared
				MRI					
Protopapas et al. (33)	1	14	Severe dysmenorrhea	MRI	Left uterine cornua	3.8	Laparoscopy	JCA	Diminishing symptoms (12 m)
Kiyak et al. (34)	1	27	Chronic pelvic pain	TVUS	Right cornual area	4.5	Laparoscopy	JCA	Diminishing symptoms (3 m)
Supermaniam et al. (35)	2	22	Severe dysmenorrhea	TAUS	Right fundic area close to the tube insertion	3.6	Laparoscopy	ACUM	Symptoms disappeared
				TR 3D-US					
				MRI					
		36	Severe dysmenorrhea	TVUS	Right intramural mass close to the round ligament insertion	3.3	Laparoscopy	ACUM	Symptoms disappeared (6 m)
Naftalin et al. (36)	8	29.2	Severe dysmenorrhea	TVUS or TRUS	N/A	2.3	Laparoscopy	ACUM	N/A
			Chronic pelvic pain						
Mollion et al. (37)	2	17	Severe dysmenorrhea	TVUS	Left uterine horn	3	Laparoscopy	ACUM	N/A
				MRI					
		23	Severe dysmenorrhea	MRI	Left uterine horn	3	Laparoscopy	ACUM	N/A
Hu et al. (38)	1	22	Chronic pelvic pain	TVUS	Left side of the myometrial	5	Laparoscopy	ACUM	Symptoms disappeared
				CT					
				MRI					
Tokgoz et al. (39)	1	17	Severe dysmenorrhea and chronic pelvic pain	TAUS	Left side of the uterus	2.5	Laparoscopy	ACUM	Symptoms disappeared (24 m)
Dekkiche	9	23	Dysmenorrhea	MRI	Left uterine wall under insertion of round ligament	1.7	Robot-assisted laparoscopy	ACUM	N/A
		19	Chronic pelvic pain	TV 3D-US	Right uterine wall under insertion of round ligament	4	Laparoscopy	ACUM	N/A
				MRI					
		18	Severe dysmenorrhea	TAUS	Left antero-fundic wall	3.8	Laparoscopy	ACUM	Diminishing symptoms (48 m)
				MRI					
		22	Chronic pelvic pain	TVUS	Right uterine wall under insertion of round ligament	2.1	Laparoscopy	ACUM	Persistent pain (48 m)
				MRI					
		35	Pelvic pain	TAUS	Right uterine wall under insertion of round ligament	2.5	Laparoscopy	ACUM	Symptoms disappeared (24 m)
				MRI					
		18	Severe dysmenorrhea	TVUS	Left uterine wall under insertion of round ligament	1.8	Laparoscopy	ACUM	Symptoms disappeared (6 m)
				MRI					
		30	Severe dysmenorrhea	TVUS	Right uterine wall under insertion of round ligament	2.4	Laparoscopy	ACUM	Persistent pain (2 m)
				MRI					
		17	Chronic pelvic pain	TVUS	Left antero-fundic wall	3	Laparoscopy	ACUM	Persistent pain (42 m)
				MRI					
		18	Severe dysmenorrhea	TAUS	Left fundic area close to the tube insertion	2.6	Laparoscopy	ACUM	Symptoms disappeared (1 m)
				MRI					
Total	87	21.89	Dysmenorrhea: 60/88 Pelvic pain: 28/88	MRI 62/88	Right: 37/88	3.3534	Laparoscopy: 73/88		Symptoms disappeared: 48/88
				US 67/88	Left: 39/88		Robot-assisted		
				Pyelography: 15/88	Central: 4/88		Laparoscopy: 2/88		Diminishing symptoms: 19/88
				HSG: 13/88	N/A: 8/88		Laparotomy: 12/88		
				CT 3/88			Op-hysteroscopy: 1/88		Persistent pain: 3/88
							Unknown procedure for 1 hysterectomy		N/A: 18/88

N/A, not available; CT, computerized tomography; JCA, juvenile cystic adenomyoma; HSG, hysterosalpingography; TVUS, transvaginal ultrasound; TRUS, transrectal ultrasound.

The clinical manifestations are always some form of pelvic pain; dysmenorrhea is the most prevalent symptom (68.2%), associated or not with CPP (31.8%).

The two most useful radiological procedures are 2D ultrasound and MRI. The latter was performed for 70.5% of the patients.

**Figure 6:**
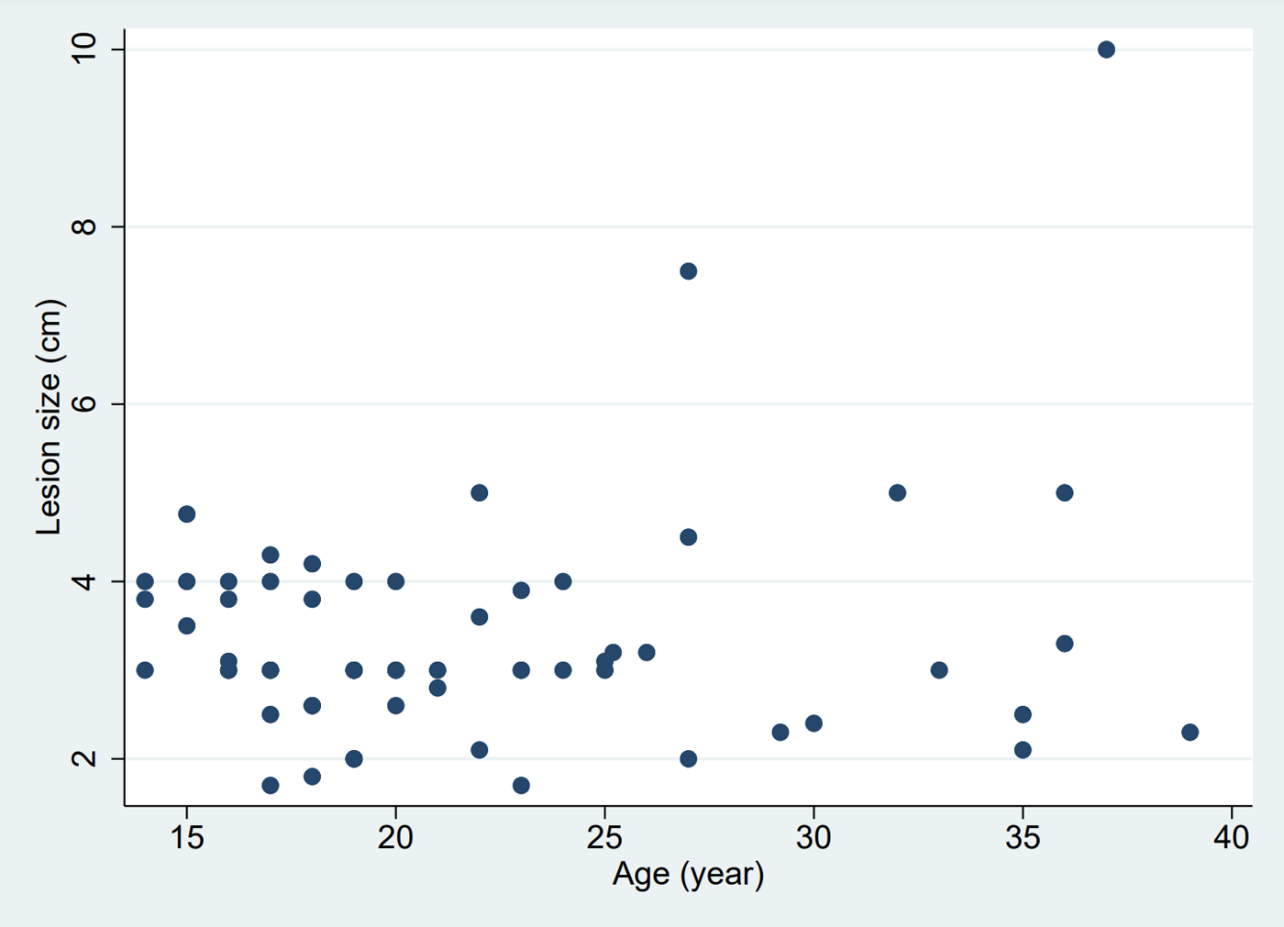
Relation between age and size of the lesion.

Usually, the mass is unique, but in rare cases, it can also be biloculated [3/88, 3.4% (26, 40)]. The lesion was lateralized 86% of the time, 42.0% right, 44.3% left, and astonishingly 4.5% were central. The mean size of the lesion was 3.4 cm. No relation between the variables “age” and “size of the lesion” was noted as shown in Figure 6. Linear regression analysis also found no relation between these two variables (*R*-squared = 0.03, *p*-value = 0.14).

Surgical resection was in 83.0% of the patients performed by laparoscopy which should be the privileged approach, in 13.6% of patients by laparotomy, in 2.3% of patients by robot-assisted laparoscopy and in 1.1% of patients by operative hysteroscopy. Clinical improvement occurred in almost all patients after surgical resection, except for a few patients (*n* = 3). To this day, no other aetiology was found for these three patients presenting persistent pain (endometriosis was excluded during laparoscopy). They are treated with conservative medical treatment.

## 4. Discussion

We consider Acién et al.'s physiopathologic hypothesis and their definition of this anomaly as the most appropriate for now and therefore decided to adopt the terminology and concept of ACUM. As opposed to some authors who consider this pathology as a focal or cystic form of adenomyosis, we do not, mainly because of its absence of recurrence, the young age of the affected patients and its pathological characteristics (point 4 of Acién's definition) which are clearly different from adenomyosis.

Regarding the age at diagnosis, which is considered for Takeuchi et al. in 2010 (17) as a diagnostic criterion when under 30, we would not be that restrictive. All the more since this diagnosis is often delayed after months or years of investigations or symptomatic treatments such as pain killers or hormonal treatments.

We want to insist on an important characteristic of an ACUM, which may not be clear enough in Acién et al.'s definition (5); the tube on the homolateral side of the lesion is always connected to the normal uterine cavity and is patent. This was already described by Takeuchi's definition of JCA (17). It also means that an ectopic pregnancy is not possible in the cavity of an ACUM. This is the principal criteria that distinguishes it from a uterine malformation type U4 (6), another rare type of Müllerian duct anomaly (also known as non-communicating rudimentary uterine horn or Robert's uterus) which is the principal differential diagnosis. It is also important to note that for now no ACUM has ever been associated with urinary tract malformation.

MRI is known as the imaging modality of choice to achieve complete exploration of female genital anomalies (41). It allows for a precise localization of the tumour and therefore helps for an appropriate curative and fertility-sparing laparoscopic resection (3). Indeed, MRI has a higher correlation with surgical findings compared with echography (42).

In case of an unclear diagnosis, complementary investigations with a hysterosalpingo-foam sonography, hysterosalpingography or per-operative chromopertubation must be performed. Fertility-preserving and non-invasive surgery is essential in these young patients.

In our experience, IHC is not mandatory for the diagnosis of ACUM, but it can help if the endometrium and the cytogenic chorion are difficult to locate on HE alone.

Salpingectomy is not indicated and definitely has to be avoided (except in the case of a coexisting tubal pathology of another ethology). Both the homolateral uterine artery and the round ligament must be preserved as much as possible. Nevertheless, if the size of the lesion is important, it can be difficult to stay minimally invasive while doing a complete resection.

Pontrelli et al. (26) described the only case of a successful hysteroscopic resection of an ACUM. This method was chosen because the MRI findings were suggestive of a bicornuate uterus with cornual hematometra in a non-communicating horn, so they planned to remove the wall of the lesion. The undeniable advantage of this technique is its short operative time and its minimal invasive character. One can question the quality of resection of the capsule which must be difficult to obtain. If this latter is incomplete, there might be a risk of recurrence. There is also the remaining issue of the future obstetrical outcome for these young patients because no sutures are made to reinforce the myometrium. This technique might also expose the patient to a higher risk of uterine rupture in case of future pregnancy than with an intra-abdominal access.

Transvaginal ultrasound-guided alcohol sclerotherapy is an interesting procedure gaining momentum in the treatment of ACUM. In 2020, the first patients was described by Merviel et al. (43) who used the same technique as for the treatment of ovarian endometriomas. In 2021, Naftalin et al. (36) reported on another four women treated with this procedure. One of them had a recurrence of symptoms 6 months after the sclerotherapy and therefore needed a laparoscopic resection. It is possible that the surgical intervention was planned due to lesion reappearance; however this is not specified by the authors. For these four patients, the diagnosis of ACUM was based on the haemorrhagic content of the mass found in cytology. As a definitive histologic diagnosis cannot be obtained with sclerotherapy, we did not include these patients in our review. This technique has several benefits over laparoscopy; it is shorter in duration, is performed under local anaesthesia, does not add an iatrogenic myometrial injury and therefore might not negatively affect the future obstetrical outcome, although information on the obstetrical risk after surgical resection of ACUM is still unknown.

While there are no reported cases of uterine rupture during pregnancy in the literature to date, one can imagine that the risk exists and is similar to that observed after an intramural myomectomy [0.93% according to Gambacorti-Passerini et al. (44)]. Patients need to be informed of this risk and be aware of it.

Finally, the incidence of ACUM is still unknown, but in the last two decades, there has been more and more literature available on this pathology, and the number of cases is increasing. This can be explained by the improvement of imaging techniques and improved knowledge of this pathology despite its rarity.

ACUM is now a well-defined uterine malformation with precise characteristics that should be known by gynaecologists and should be evoked in the differential diagnosis of severe dysmenorrhea and CPP. The decision of whether a conservative or a surgical therapy should be done has to be made with the patient according to their preferences. Long-term outcome for these patients is still unknown and has to be especially studied regarding the potential recurrence of the lesions and the obstetrical outcomes. Hysteroscopic resection and ethanol sclerotherapy are two new interesting therapeutic approaches that need to be explored in the future to treat ACUM.

ACUM is certainly underdiagnosed, because it is a poorly known pathology hardly ever researched in a context of acute and early dysmenorrhea. With our cases, we also want to stress that ACUM has to be thought of and looked for in the case of atypical, chronic pelvic pain, in pre-menopausal women.

Concerning the limitations of this study, we would highlight its retrospective character. Moreover, the heterogeneous qualitative description of the cases found in the literature makes the comparison between them difficult and limits the potential of meaningful statistical analysis. Finally, as ACUM is a rare pathology, the number of studied cases is relatively small, which makes its understanding still incomplete.

## Data Availability

The original contributions presented in the study are included in the article/Supplementary Material, further inquiries can be directed to the corresponding author.
